# Shifting Spike Times or Adding and Deleting Spikes—How Different Types of Noise Shape Signal Transmission in Neural Populations

**DOI:** 10.1186/2190-8567-5-1

**Published:** 2015-01-12

**Authors:** Sergej O Voronenko, Wilhelm Stannat, Benjamin Lindner

**Affiliations:** Bernstein Center for Computational Neuroscience, 10115 Berlin, Germany; Department of Physics, Humboldt University, 12489 Berlin, Germany; Institut für Mathematik, TU Berlin, 10587 Berlin, Germany

**Keywords:** Time-dependent input, Population coding, Common noise, Shifting of spikes, Addition and deletion of spikes, Mutual information, Suprathreshold stochastic resonance

## Abstract

We study a population of spiking neurons which are subject to independent noise processes and a strong common time-dependent input. We show that the response of output spikes to independent noise shapes information transmission of such populations even when information transmission properties of single neurons are left unchanged. In particular, we consider two Poisson models in which independent noise either (i) adds and deletes spikes (AD model) or (ii) shifts spike times (STS model). We show that in both models *suprathreshold stochastic resonance* (SSR) can be observed, where the information transmitted by a neural population is increased with addition of independent noise. In the AD model, the presence of the SSR effect is robust and independent of the population size or the noise spectral statistics. In the STS model, the information transmission properties of the population are determined by the spectral statistics of the noise, leading to a strongly increased effect of SSR in some regimes, or an absence of SSR in others. Furthermore, we observe a high-pass filtering of information in the STS model that is absent in the AD model. We quantify information transmission by means of the lower bound on the mutual information rate and the spectral coherence function. To this end, we derive the signal–output cross-spectrum, the output power spectrum, and the cross-spectrum of two spike trains for both models analytically.

## 1 Introduction

Neurons in the sensory periphery encode information about continuous time-dependent signals in sequences of action potentials. Hereby, upon repeated presentation of a stimulus, the response of the neuron is not perfectly reproducible but exhibits trial-to-trial variability. Processes, leading to such variability, are termed *noise* and can have various origins [1, 2]. How such noise processes affect the transmission of time-dependent signals in neurons can be studied in the framework of information theory [3, 4]. Within this framework, it has been shown, for instance, that the presence of noise can enhance the transmission of weak (subthreshold) signals in single neurons and neural models [5–7], an effect known as *stochastic resonance* and also observed outside biology [8, 9]. At the level of neural population coding, noise can also have a beneficial role for the transmission of strong (suprathreshold) signals [10, 11] by means of *suprathreshold stochastic resonance* (SSR), the mechanism of which is quite distinct from that of conventional stochastic resonance despite the similarity in their naming. Additionally, noise not only impacts the total transmitted information, but it also affects which frequencies of the sensory signal are preferably encoded by a neural system. The suppression of information about the input signal in certain frequency bands can be regarded as a form of information filtering [12–16]. Put differently, we may ask whether the neural system is preferentially encoding slow (low-frequency) components of a signal or fast (high-frequency) components of a signal, which can be quantified by the coherence function, as described below.

**Fig. 1:**
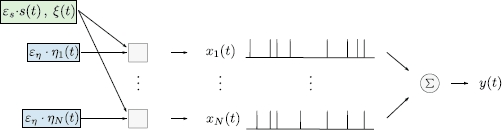
A population of *N* uncoupled neurons is driven by common processes (*green box*) and independent noise processes (*blue boxes*). The common processes consist of a strong common noise  and a common sensory signal , which is multiplied with a small positive scaling parameter . Independent noise processes  are multiplied with another small positive scaling parameter . In the special case of ,  the sensory signal is absent and the *N* neurons exhibit spontaneous activity. Due to the strong common noise *ξ* the spontaneous activity is highly correlated. In the special case of ,  all neurons generate identical output spike trains encoding the sensory signal in the time-dependent firing rate. The output of the population is quantified by the sum  of the individual spike trains

How noise affects information transmission in neural populations has been studied for a long time [11, 17, 18]. Of particular interest in the context of the information flow through a population are the correlations among neurons that have been observed in many experimental preparations, e.g. in the visual system [19–22], the somatosensory system [23], the olfactory system [24, 25], the barrel cortex of rats [26, 27], and in spinal motor neurons [[28], and references therein]. Such correlations, either in membrane potential, in output spikes, or in spike counts of two cells, can be caused by a common input to both cells due to overlapping receptive fields. For instance, in the electrosensory system [29], the spontaneous activity of different neurons in the absence of the signal is uncorrelated and is driven by independent noise processes. In other systems, the output correlations are not caused by a stimulus. For example, in tangential neurons of the fly visual system, already the noise processes are correlated and lead, even in the absence of the sensory signal, to a spontaneous spiking activity that is correlated across different neurons [20] (for a detailed discussion of the noise sources see [30]). Other examples of neurons receiving common noise input are ganglion cells of the primate retina [21] or the projection neurons of the *Drosophila* olfactory system [25]. In the present study, we consider ensembles of neurons receiving highly correlated noise input as sketched in Fig. 1.

**Fig. 2:**
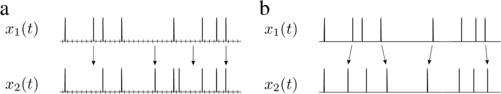
Two neurons are driven by a strong common noise *ξ*, a weak common signal *s*, and independent noise processes . **a** Addition and deletion of spikes: Independent noise processes lead to addition and deletion of spikes by weakly modulating the threshold value  in Eq. (11) independently for both neurons. *The first arrow* indicates the deletion of a spike, *the second arrow* indicates the addition of a spike, and *arrows three and four* indicate time bins where there is no change in the spike trains. **b** Spike time shifting: Independent noise leads to shifting of spikes by weakly modulating the integrand  in Eq. (16) independently for both neurons, but no spikes are added or deleted. *The arrows* exemplify corresponding spikes in the two spike trains that have been shifted in time

We consider two theoretical models of neural populations that exhibit strong spike train correlations among the neurons within the population, even in the absence of a sensory signal. In this situation, we address the question of how the spike trains of different neurons may be decorrelated by independent noise processes and how this affects the transmission of a sensory signal. More specifically, we are interested in how independent noise influences the spikes of the output spike trains and study two extreme cases. In one case, we assume that independent noise adds and deletes spikes in the output spike trains (AD model) as illustrated in Fig. 2a. This is a likely effect of additional noise in an excitable neuron with low firing rate. In another case, we assume that independent noise shifts the spike times of the output spike trains (STS model) as illustrated in Fig. 2b. This scenario applies to neurons in a tonically firing regime, which generally do not fire with Poisson statistics. We construct the two models in such a way that they cannot be distinguished on a single neuron level. This allows us to ascribe any differences in the information transmission properties of the populations unambiguously to the different effects of the noise.

This work is organized as follows: First, we describe the methods by which we will study the effect of noise on signal transmission in a population of spiking neurons. Second, we introduce two models where independent noise either adds and deletes spikes, or shifts spike times in the output spike trains. In Sect. 4, we then derive the spectral statistics for the two models. These derivations can be skipped upon the first reading. In Sect. 5, we summarize the derived spectral statistics and proceed to study the effect of independent noise on information filtering and the total transmission of information in neural populations. We conclude with a summary and a discussion of our results in Sect. 6.

## 2 Methods

### 2.1 Spike Train Statistics & Ensemble Averages

In this paper, we study the transmission of a sensory time-dependent signal by a population of spiking neurons, which is illustrated in Fig. 1. We model the output spike trains of single neurons by stochastic point processes. The output of the *μ* th stochastic point process can be described by the spike count . This function starts at 0 at  and is incremented by 1 at each spike time , i.e.  for ,  for , and so forth. Equivalently, the output of a stochastic point process can be described by the derivative of . This derivative is called the spike train and is given by a sum of delta functions, 1

We study information transmission properties of the population by quantifying the amount of information about the input signal  encoded in the sum 2

of the individual output spike trains.

We take into account different sources of variability: common noise , independent noise sources , and the stochastic signal  (cf. Fig. 1). Consequently, we can consider different ensemble averages, denoted by angular brackets . Subscripts indicate over which processes we average and the absence of subscripts implies averaging over all involved processes. In mathematical terms this notation corresponds to the expectation with respect to the conditional distribution that is indicated by the subscripts, e.g.  stands for the expectation of the process  with respect to the conditional distribution of *ξ*, conditioned on a realization of *s* and *η*, whereas  stands for the total expectation. Note that  is still a random process, unless a realization of *s* and *η* is fixed. Below, when analyzing correlation functions, e.g. Eq. (7), we will also consider averages over products of spike trains , which in mathematical terms corresponds to 

This applies analogously to averages over the processes  and .

The instantaneous firing rate 

obtained by averaging the spike train *only* with respect to the common noise , will be an important quantity in our calculations. It still depends on the independent noise and the signal and is difficult to determine in experiments. More accessible is the average over all noise sources by repeated trials with a frozen stimulus and summation over all spike trains. In this way, we obtain (apart from a normalization factor ) the population rate 3

**Fig. 3:**
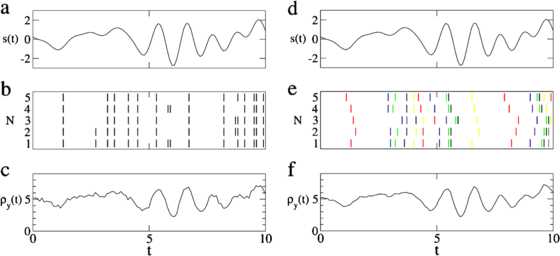
Simulations of the population response for the AD model (**a**–**c**) and the STS model (**d**–**f**). Panels **a** and **d** show the same realization of an input signal. Panels **b** and **e** show 5 neurons of a population for the two models subject to the signal realization from panels **a** and **d** and the same noise realizations (not shown). In the AD model (panel **b**) independent noise leads to addition and deletion of spikes. In the STS model (panel **e**) independent noise leads to shifting of spikes, as indicated by *the coloring*. Panels **c** and **f** show the population response Eq. (3) to the signal shown in **a** and **d**, averaged over 5000 realizations of the noise processes. The model parameters were , , ,

An example for a signal, spike trains, and the resulting population rate is shown in Fig. 3.

### 2.2 Information Transmission & Spectral Statistics

In the case of ergodic processes, the total amount of the information about a signal  transmitted by the output  can be quantified by the *mutual information rateR* [3], which is measured in bits per second. For Gaussian signals, a lower bound on the mutual information rate [4, 31, 32] is given by 4

The coherence function  between the input signal  and the output  is calculated from second-order spectral measures of input and output and is defined as 5

Here  and  are the summed-spike-train and signal power spectra, respectively, and  is the signal–output cross-spectrum. The numerical estimation of spectra follows standard procedures [33]. In our analytical calculations we will use the Wiener–Khinchin theorem [34]6

that relates the spectra to the correlation functions in the time domain 7

The limit of large times in Eq. (7) ensures stationarity. For the summed spike train Eq. (2), the autocorrelation function with  in Eq. (7), can be rewritten as 8

where  is the spike train autocorrelation function and  the cross-correlation function between two spike trains. Analogously, the signal–output cross-correlation function can be written as 9

where  is the cross-spectrum between the input signal and a single output spike train. Taking the Fourier transformation of Eqs. (8) and (9), using the Wiener–Khinchin theorem Eq. (6), and inserting the results into Eq. (5), yield the coherence function 10

From Eq. (10) we see that for  the cross-spectrum of two spike trains, , appears in the denominator of the coherence function and gains significance as *N* becomes larger. Therefore, an essential theoretical problem is to calculate this cross-spectrum.

As outlined above, the coherence function allows one to estimate the total flow of information through the neural population. However, because  enters in a monotonic fashion in Eq. (4), we can also regard the coherence as a frequency-resolved measure of information transfer. Reduction of the coherence in certain frequency bands can be regarded as a form of information filtering, which needs to be distinguished from power filtering. Hence, besides the lower bound , we will also inspect the frequency dependence of the coherence function.

## 3 Models

The models that we consider in this paper have the following assumptions in common:

1. Poisson statistics of spontaneous activity;
2. high correlations among neurons due to strong common noise input;
3. encoding of a sensory signal in the time-dependent population rate.

For simplicity, we consider a linear encoding of a weak time-dependent signal. This will allow us to use the lower bound on the mutual information rate as an approximation for the total transmitted information. Note that, although already a single Poisson process can show conventional stochastic resonance [35], with our linear encoding paradigm we exclude this possibility. In our models, the signal transmission in a single neuron is always degraded by noise.

In our theoretical model, we assume that all neurons fire to zeroth-order in complete synchrony and a weak noise input, which is independent for every neuron, leads to a decorrelation of the output spike trains. For simplicity, we assume that for each neuron the independent noise process and the sensory signal are additive. Both, the sensory signal and the independent noise signals, are modeled by Gaussian processes with unit variance and zero mean.

The considered models can be regarded as *inhomogeneous Poisson processes* [36], which are rate-modulated by a common signal  and an independent noise . Such processes are examples of a *doubly stochastic process* [37] or a *Cox process* and are a special case of the *inhomogeneous Bernoulli process* [38]. The simplicity of the considered models will allow us to characterise the information transfer of weak time-dependent signals analytically. Note that the assumptions (1)–(3) made above describe, in good approximation, spiking in specific sensory systems, e.g. in tangential neurons of the fly visual system [20, 39, 40]. The additional modifications that make up the differences between our two models can be regarded as additional operations on the spike trains in the form of thinning (or the opposite of it) and the introduction of an operational time [37, 41].

Before we introduce in detail the two models sketched in Fig. 2, it is worth to note that, for weak stimuli and weak independent noise, these models possess the same signal–output cross-spectrum , the same power spectrum , and the same time-dependent output firing rate. Therefore, for  the coherence function and the information rate are identical for both models. The models are mainly distinguished by how independent noise affects the spikes of the output spike trains, which results in different cross-spectra  of two spike trains. This setup allows us to study how the response of spikes to noise affects information transmission in neural populations, while keeping all other potential influences on signal transmission unchanged.

### 3.1 Addition and Deletion Model (AD Model)

In the following, we introduce the model for a population of spiking neurons where independent noise adds or deletes spikes. First, we discretize the time axis into bins of width Δ*t*. We generate a spike in the *j* th time bin in the *μ* th spike train, whenever the following condition is fulfilled: 11

The common noise process *ξ* is uniformly distributed in  and uncorrelated in time. The spikes are assigned the height  such that the discrete spike train reads 12

where  is the Heaviside function (implementing the indicator function) and the second argument of  indicates the time-discretized version of the spike train. Here  is the midpoint of the time bin where the *k* th spike of the *μ* th spike train was generated. In the limit  the spike train  approximates the sum of *δ*-functions  given by Eq. (1).

We can compute the ensemble average of the spike train over the common noise *ξ*13

The average  is conditioned on specific realizations of the processes *s* and . As we show explicitly in Appendix A in Eq. (57), averaging additionally over the independent noise and the signal, one finds in the limit of 14

Throughout the paper, we will consider the limit , such that we can neglect correction terms like the one in the above equation.

In the left column of Fig. 3, we show how a sensory signal is encoded in the population firing rate  of a population of five AD neurons and how the output spike trains of the neurons are modulated by independent noise.

### 3.2 Spike-Time-Shifting Model (STS Model)

Next, we introduce the model for a population of spiking neurons where independent noise shifts the spike times of the output spike trains. To zeroth-order the *N* neurons of the population generate identical spike trains 15

which we model by a homogeneous Poisson process with mean firing rate  and spike times . For the *μ* th neuron, the times  are transformed into new spike times  via the transformation 16

with  defined in Eq. (11). For a given spike time , we integrate the right hand side of Eq. (16), until the integral attains the value  [36]. The resulting integration boundary  is then the *k* th spike time of the *μ* th spike train . In general, due to the different independent noise processes , the output spike trains  will be different for each neuron. Hereby, each spike train is an inhomogeneous Poisson spike train with a time-dependent firing rate. The procedure described in this section is equivalent to the simulation of a perfect integrate-and-fire neuron with exponentially distributed thresholds [36]. The time *t* obtained after the transformation of the time axis *h* in Eq. (16) is also known as operational time [37, 41].

Although we do not model the underlying noise process explicitly, we think of the homogeneous spike trains in Eq. (15) as a result of a common noise process *ξ*, analogously to the AD model. By the average , we will denote the average over different realizations of the homogeneous Poisson spike trains in Eq. (15).

For a homogeneous Poisson spike train that is transformed according to Eq. (16) with , the average over the spike train for a fixed realization of the signal and the independent noise reads  [36]. For a process  that is not bound by zero this is not strictly fulfilled. Hence, ensemble averages over the spike train will contain correction terms that are proportional to the square root of the probability that  is smaller than zero, which we calculated in Appendix A in Eq. (52). Consequently, using Eq. (11), we obtain for the averaged spike train 17

which in the limit  leads to the same mean firing rate as for the AD model Eq. (14) in the limit of .

A simulation of five spike trains of the STS population, driven by a common noise process *ξ*, a common signal *s*, and independent noise processes , is shown Fig. 3e. Note that the modulation in Eq. (16) is very distinct from adding jitter to the single spike times, as is considered in [42–44], in that the modulation of the spike times presented here preserves the order of the spikes in each spike train. Other models that incorporate the deletion of spikes in a Poisson spike train [45] or a combination of deletion and shifting as in the thinning and shifting model [42, 44], differ from the models presented here in that the single spike trains of those models are homogeneous spike trains with constant rates. However, the models in the present paper are designed such that the single spike trains have a prescribed time-dependent firing rate , which still depends on the realization of the signal *s* and the individual noise *η*. The cross-correlations between spike trains are a consequence of the different implementations of the time-dependent firing rate and are not prescribed a priori as in [42, 44, 45]. Even if the deletion or shifting of spikes in the thinning and shifting model is performed on a rate-modulated mother process, the resulting process would not be equivalent to the AD model or STS model, in which the addition and deletion of spikes and the shifting of spike times are not independent of the signal realization. In particular, the thinning and shifting model of a population of daughter processes for which the stimulus is solely encoded in the firing rate of the mother process cannot exhibit suprathreshold stochastic resonance.

### 3.3 Modeling the Common Signal and the Independent Noise Processes

The sensory signal *s* and the independent noise sources  are modeled by Gaussian stochastic processes with zero mean and unit variance. For simplicity, we choose for both, the signal and the independent noise, a flat power spectrum, 18

where  and  are lower and upper cutoff frequencies, respectively. Throughout the paper, we will consider a finite upper cutoff frequency and a non-vanishing lower cutoff frequency. As we will show in our analytical calculation below, the cross-spectrum for two spike trains of the STS model is finite only for . A realization of the common signal *s* is shown in Fig. 3a and 3d.

### 3.4 Simulations

In contrast to the AD model, the numerical measurement of the statistics of the STS model requires a careful choice of simulation parameters. Depending on the shape of the cross-spectrum between different spike trains for the STS model, one has to choose a large simulation time to ensure stationarity and a very small time discretization to be able to resolve correlations between spike trains on small time scales. Furthermore, the coherence function systematically depends on the number of realizations used for the numerical averaging of the spectral statistics.The values of the time discretization Δ*t*, the total simulation time *T*, and the number of realizations  used for the numerical averaging of the spectral statistics are reported in Table 1.

**Table 1:** **Parameters used in numerical simulations**

Figure	Δ*t* in seconds	*T* in seconds	
Fig. 3	1⋅10^−1^	1⋅10^3^	5⋅10^3^
Fig. 4	5⋅10^−6^	1⋅10^2^	5⋅10^2^
Fig. 5	1⋅10^−4^	1⋅10^2^	5⋅10^4^
Fig. 6a	5⋅10^−5^	3⋅10^1^	1⋅10^5^
Fig. 6b	3⋅10^−5^	2⋅10^2^	1⋅10^4^
Fig. 6c	1⋅10^−4^	3⋅10^2^	1⋅10^4^
Fig. 6d	3⋅10^−5^	1⋅10^3^	2⋅10^3^
Fig. 7a STS	5⋅10^−5^	2⋅10^2^	2⋅10^4^
Fig. 7a AD	5⋅10^−5^	3⋅10^1^	1⋅10^5^
Fig. 7b STS	5⋅10^−5^	1⋅10^3^	2⋅10^3^
Fig. 7b AD	1⋅10^−4^	3⋅10^2^	1⋅10^4^
Fig. 8	2⋅10^−4^	1⋅10^3^	2⋅10^4^
Fig. 9	2⋅10^−4^	1⋅10^2^	–

## 4 Derivation of Spectral Measures

### 4.1 Input–Output Cross-spectrum

In this section, we calculate the spectral measures that are necessary to quantify information transmission properties of the populations. We start by considering the input–output cross-correlation function 

The correction terms can be derived in complete analogy to the calculation in Appendix A. The first term in the above equation can be calculated using Eq. (11) and the fact that *s* and  are Gaussian processes with unit variance and zero mean, which leads to 

In the limit , keeping only the first-order term in , the correction term in the above equation can be neglected. Then, after a Fourier transformation, we find the input–output cross-spectrum 19

which is equal for both models.

### 4.2 Cross-spectrum for Two Spike Trains for the AD Model

The cross-correlation function between two spike trains is defined as 20

where  and  are different spike trains of a population with . The ensemble averages in the above equation are taken over four stochastic processes: The common noise *ξ*, the common signal *s*, and the independent noise processes  and . Employing Eq. (14), we can write the second term in Eq. (20) as 

The first term in Eq. (20) can be interpreted as a probability density [4]. Choosing a discrete variant of the spike train  as introduced in Eq. (12), this leads to 

(Pr stands for probability and ST stands for spike train). As we generate the spike trains in discrete time steps, we first consider the cross-correlation function between two spike trains with a finite time discretization  with  . Splitting the expression in the above equation into two parts, one for  and one for , we obtain 21

with 

and 

Note that, due to stationarity of the stochastic signals and spike trains, the probabilities in Eq. (21) do not depend on *t*. As described in Sect. 3.1, the values of realizations of the process *ξ* at different times are independent of each other, which allows us to average both spike trains separately leading to 

Using Eq. (11) in the above equation and employing that  and  are independent Gaussian processes with zero mean we obtain in the limit 22

From the definition of the AD model in Eq. (11), we can infer that the probability of observing a synchronous spike in two spike trains equals the probability that the thresholds  and  are both higher than the realization of the common noise variable . Then, dropping the time arguments, the probability of synchronous spiking can be expressed as an average over two theta functions 23

As is shown in Eq. (59) in Appendix B, we can write the above expression for weak sensory signals as 24

Inserting Eqs. (22) and (24) in Eq. (21), and taking the limit , we obtain 

and for the cross-correlation function between two spike trains Eq. (20) we find 

In the limit , keeping terms up to second-order in  and , we can neglect the correction terms in the above equation and find the following cross-spectrum between two spike trains: 25

**Fig. 4:**
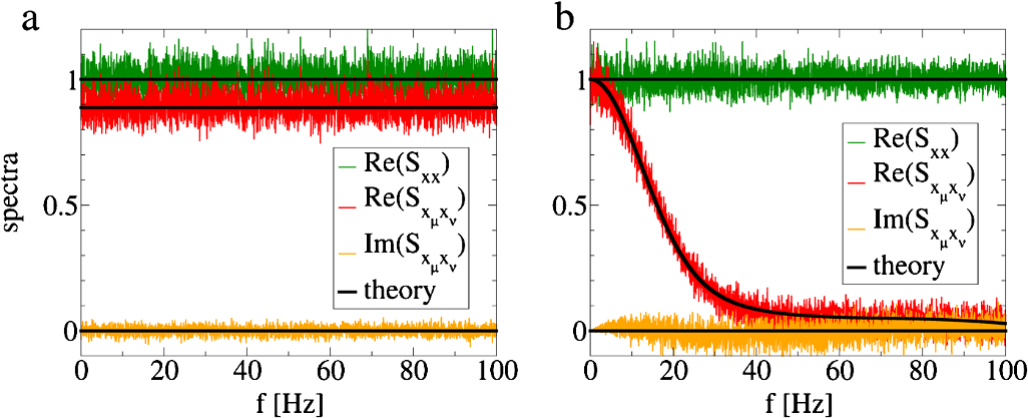
**a** AD model: Simulation results of the spike train power spectrum (*green*) and the real and imaginary part of the cross-spectrum of two spike trains (*red* and *yellow*, respectively) are compared with the analytical results Eq. (39) and Eq. (25) (*black lines*). **b** STS model: Simulation results of the spike train power spectrum (*green*) and the real and imaginary parts of the cross-spectrum of two spike trains (*red* and *yellow*, respectively) are compared with the analytical results Eq. (39) and Eq. (38) (*black lines*). The model parameters were , , , ,

The above equation shows that by adding and deleting spikes the weak independent noise sources lead to a decorrelation of the two spike trains with a uniform decrease of power at all frequencies proportional to . The analytical result for the cross-spectrum of two spike trains Eq. (25) for the AD model is compared with simulations in Fig. 4. Note that because the cross-correlation function between two spike trains is symmetric with respect to *τ*, the cross-spectrum is real-valued for all frequencies.

### 4.3 Cross-spectrum for Two Spike Trains for the STS Model

In this section, we calculate the cross-spectrum  between spike trains *μ* and *ν* for the STS model. We first consider the autocorrelation function 

of a homogeneous Poisson process with constant rate , where we use a slightly different notation than in Eq. (7). The last term in the above equation equals . The spike trains inside the first average can be expressed as derivatives of the spike count  as in Eq. (1), such that 26

The power spectrum of a homogeneous Poisson process is constant  and implies for the autocorrelation function of a homogeneous Poisson process 27

Combining Eq. (27) with Eq. (26), we obtain 28

Now we calculate the cross-correlation function between two spike trains 29

of the full process, subject to an intrinsic noise *ξ*, independent noise processes  and , and an input signal *s*. Employing Eq. (17), the last term in the above equation can be written as 30

The first term of the cross-correlation function can be recast as before into 31

The rate-modulated Poisson process generated by the STS model is related to a homogeneous Poisson process with constant rate by the time transformation Eq. (16). We use this relation to link the inhomogeneous to the homogeneous spike count via 32

Using the above relation and Eq. (28), we find 

Note that the above relation is valid only if  is strictly larger than zero. Hence, we obtain for Eq. (31) 33

where the correction term is proportional to the square root of the probability that  computed in Eq. (52). Using , employing the relation Eq. (62) derived in Appendix C, and substituting the variables  and , we transform Eq. (33) into 34

Using the definition of  Eq. (32), we can write the average over the delta function in Eq. (34) as 

The new stochastic variable *g* is a sum of two integrals over Gaussian variables and therefore also a Gaussian variable. The average of the delta function over realizations of *g* is then the probability that *g* attains the value , and is given by 35

where  is the variance of . In Appendix D, Eq. (64), we show that 36

for our specific choice of a flat noise power spectrum, introduced in Sect. 3.3. Employing Eqs. (35), (34), and (30) in Eq. (29) and expanding up to second-order in , we obtain for the cross-correlation function for two spike trains 37

We note that the linear term in  vanishes due to the zero mean of the Gaussian signal . Equivalently, all higher-order odd terms in  in Eq. (37) vanish due to the Gaussian nature of the signal (except for the correction term due to realizations of signal and individual noise that lead to ). From Eqs. (36) and (37) it can be seen that for a vanishing lower cutoff frequency of the independent noise spectrum (), the variance  diverges and as a consequence of this the cross-correlation between the two spike trains vanishes—only the part that is due to the signal (second term in Eq. (37)) still contributes.

After Fourier transforming Eq. (37) (neglecting the correction terms), we find the cross-spectrum for two spike trains in the STS population, 38

In Fig. 4, the analytical result for the cross-spectrum for two spike trains of the STS model Eq. (38) is compared with simulations. As for the AD model the cross-spectrum of two spike trains is real valued. In contrast to the AD model Eq. (25), the cross-spectrum of two spike trains for the STS model Eq. (38) exhibits a strong decrease at high frequencies, while it approaches the spike train power spectrum Eq. (39) at low frequencies. Note that, although we derived  only up to second-order in , the theory fits the simulation results very well even for .

### 4.4 Single Spike Train Power Spectrum

In Appendix E in Eqs. (67) and (72), we derive the spike train power spectrum which in the limit of  and  (keeping terms up to second-order in  and ) is equal for the AD and STS model 39

For  and , the power spectrum is flat, as we would expect for homogeneous Poisson spike trains.

## 5 Information Transmission in Neural Populations

Here, we use the spectral measures derived in the previous section to study information transmission in two neural populations. The populations are constructed in such a way that they both encode the sensory signal in the time-dependent population firing rate, and both exhibit identical single-spike-train power spectra and identical signal–output cross-spectra. The main difference between the populations lies in the effect that independent noise has on the spikes of the output. In one population independent noise adds and deletes spikes (AD model), while in the other independent noise leads to spike-time-shifting (STS model). We quantify the total of the transmitted information about the sensory signal via the lower bound on the mutual information rate Eq. (4), 

and study information filtering by means of the coherence function Eq. (10), 

The input–output cross-spectrum Eq. (19) and the single spike train power spectrum Eq. (39) read 

while the different cross-spectra between two spike trains for the two different models are given by Eq. (25), and Eq. (38): 

In all expression above, we considered the limits  and . If the sensory signal is weak compared to the noise processes driving the neurons, as is assumed throughout this paper, the coherence is much smaller than one. This allows us to employ an approximation for the lower bound on the mutual information rate, 40

in the analytical calculations to obtain simpler expressions. In the subsequent sections, we will study information transmission in populations of AD neurons and STS neurons.

### 5.1 AD Population

Inserting the single spike train power spectrum Eq. (39), the input–output cross-spectrum Eq. (19), and the cross-spectrum for two spike trains Eq. (25) into Eq. (10), we find for the coherence function of the AD population 41

**Fig. 5:**
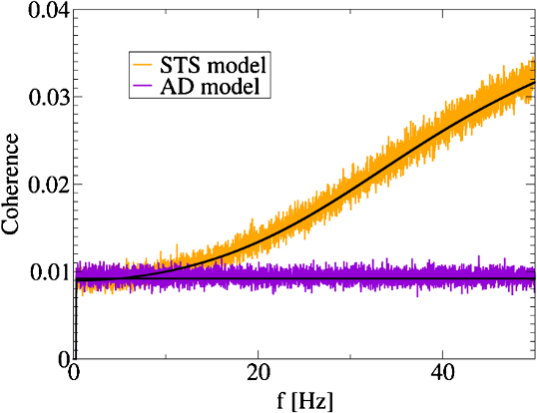
Simulation results for the spectral coherence function for the AD model (*indigo*) and the STS model (*orange*) for  and a comparison with analytical results Eq. (41) and Eq. (46) (*black lines*). The AD population exhibits a flat coherence function and corresponds to a broadband filter of information. The coherence function of the STS population is monotonously increasing with the frequency and leads to a high-pass filter of information. The model parameters were , , , , , and

Here, we used that signal and noise have equal power-spectra , as described in Sect. 3.3. The coherence function for the AD model is plotted and compared with numerical simulations in Fig. 5. The only dependence of the coherence function Eq. (41) on frequency comes from the signal power spectrum . Therefore, for a flat signal power spectrum the coherence function of the AD model is also flat for frequencies . Consequently, a population of AD neurons can be referred to as a broadband filter of information, because the sum of the output spike trains contains equal amounts of information about different frequency bands of the signal.

Inserting the coherence Eq. (41) into Eq. (40) and employing Eq. (18), we obtain for the lower bound on the mutual information rate of the AD population 42

**Fig. 6:**
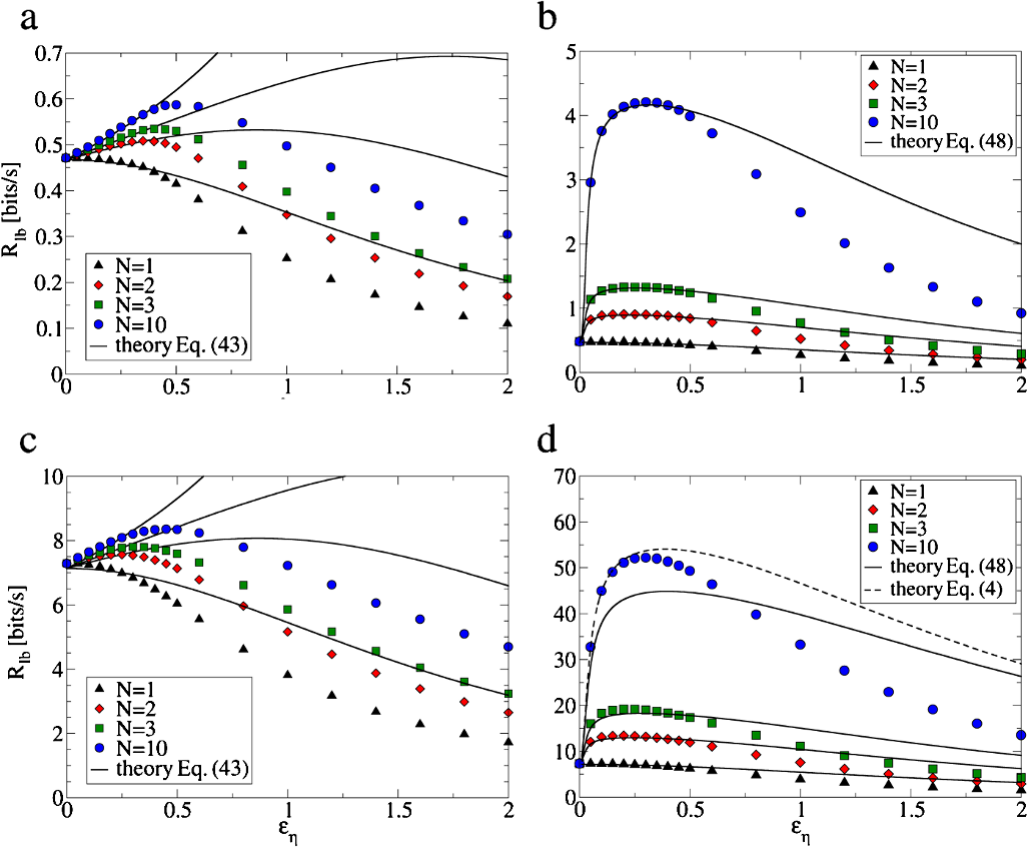
The lower bound on the mutual information rate  for the AD population (**a**, **c**) and the STS population (**b**, **d**) as a function of the independent noise level . The analytical results Eq. (42) and Eq. (47) (*black lines*) are compared with numerical simulations (*circles*) of the lower bound on the mutual information rate for various *N* for a weak sensory signal with  (panels **a**, **b**) and a stronger sensory signal with  (panels **c**, **d**). For  the lower bound on the mutual information rate is identical for both models and is degraded by independent noise. For  both models exhibit SSR, where a non-vanishing level of independent noise increases the lower bound on the mutual information rate. Hereby, the STS population profits significantly more from independent noise than the AD population. For  in panel **d** the theory Eq. (47) fails due to the linearization of the logarithm in Eq. (40). Here we plot Eq. (4), where we inserted the analytically calculated coherence function Eq. (46) and integrated numerically. We note that our analytical theory appropriately describes the increase of the mutual information for weak independent noise levels. The firing rate was  and the signal and noise cutoff frequencies were  and

The approximate expression Eq. (42) is compared with simulations for two sets of parameters in Fig. 6.

For , the last term in the denominator of Eq. (42) vanishes and the lower bound of the mutual information rate can be simplified as 43

From the above equation, it becomes evident that in a single neuron an increase of the independent noise level can only decrease the lower bound on the mutual information rate. For , additional independent noise () has a positive effect on information transmission and SSR is observed. The denominator of Eq. (42) is a quadratic function in  and exhibits a minimum at a finite level of independent noise, resulting in a maximum of the lower bound on the mutual information rate. To study the behavior of  for weak independent noise, we expand Eq. (42) with respect to  and obtain 44

with 45

The linear term in Eq. (44) is always positive. Hence, the population of AD neurons always profits from weak independent noise regardless of the specific choice of model parameters.

### 5.2 STS Population

Inserting the single spike train power spectrum Eq. (39), the input–output cross-spectrum Eq. (19), and the cross-spectrum for two spike trains Eq. (38) into Eq. (10), we find for the coherence function of the STS population 46

where  and  are defined in Eq. (38). As for the AD model discussed above, we used that signal and noise have equal power-spectra . Due to the frequency dependence of the cross-spectrum , the coherence function also depends strongly on the frequency and exhibits a monotone increase as shown in Fig. 5. Thus, the population of STS neurons can be regarded as a high-pass filter of information, similar to that observed for heterogeneous short-term plasticity [16] or coding by synchrony [13, 15].

In order to understand the high-pass filter effect in the coherence function as well as the stochastic resonance effect discussed below, we note that the cross-correlations between different spike trains contribute largely to the sum’s output variability, in particular in the absence of intrinsic noise. This output variability is quantified by the output’s power spectrum and appears in the denominator of the coherence function. With individual intrinsic noise, spike times of different neurons are slightly shifted, drastically reducing cross-correlations at high frequencies and thus the amount of the signal-unrelated variability in these frequency bands. Therefore, the coherence function increases with frequency.

Inserting Eq. (46) into Eq. (40) and inserting the noise and signal power spectrum Eq. (18), we find for the lower bound on the mutual information rate of the STS population 47

The lower bound on the mutual information rate for the STS population is compared with simulations for two sets of parameters in Fig. 6. We observe that for the given parameters the STS model shows a large SSR effect, while the AD model profits only weakly from additional noise.

**Fig. 7:**
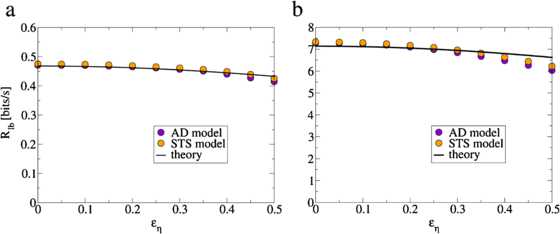
Comparison of the lower bound on the mutual information rate between the AD model (*indigo*) and the STS model (*orange*) for  for a weak signal with  (panel **a**) and a stronger signal with  (panel **b**). The analytical prediction Eq. (43) (*black line*) is in good agreement with numerical simulations (*colored points*). As predicted by our theory, a single neuron transmits the same amount of information in both models. For strong independent noise and a strong sensory signal we observe a slight deviation of the theory from simulation results, as well as slight differences between the two models. These differences are due to nonlinear effects of the independent noise and signal on the time-dependent firing rate. Model and simulation parameters were as in Fig. 6

For , the frequency dependent term in the integrand of Eq. (47) vanishes and the lower bound on the mutual information rate transforms into 

which is equal to Eq. (43) for the AD model. We compare the lower bound on the mutual information rate for  for the two models numerically in Fig. 7 for different signal strengths and independent noise levels. For sufficiently low levels of independent noise, there is no difference in the amount of transmitted information for the AD and the STS model on the level of a single neuron. By construction, from the observation of one single spike train it is impossible to distinguish between the two models.

For , additional noise can have a positive effect on information transmission, as illustrated in Fig. 6. Increasing  leads to a decrease of  in the denominator of the integral Eq. (47), as already discussed in the beginning of this section in the context of the high-pass coherence function. However, an increase of  also increases the second term in the denominator of Eq. (47), which is proportional to . Therefore, whether SSR is observed depends on the specific parameter values chosen. As for the AD model, we expand the lower bound on the mutual information rate Eq. (47) with respect to  and obtain 48

with  defined in Eq. (45). The above expansion illustrates that, when the independent noise vanishes, the lower bound on the mutual information rate is identical for the two models for arbitrary *N*. The second-order term in Eq. (48) can attain both negative and positive values depending on the choice of the model parameters. The condition that the second-order term becomes negative and that the lower bound on the mutual information rate at  is a decreasing function of  reads 

**Fig. 8:**
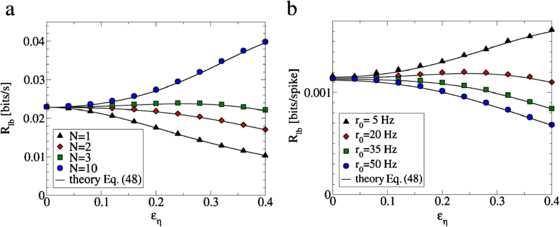
The presence of SSR in the STS population is parameter dependent, in contrast to the AD population where SSR is always observed for . **a** The lower bound on the mutual information rate for the STS population is plotted for various population sizes as a function of the individual noise level . SSR is observed for  but not for . The model parameters were , , , and . **b** The lower bound on the mutual information rate for the STS population is plotted in units of bits per spike for various mean firing rates . SSR is observed for  but not for . The model parameters were , , , and

If the above condition is fulfilled, the weak individual noise does not improve the information transmission of a sensory signal and no SSR is observed. Two examples are shown in Fig. 8. In contrast to the AD model, where SSR is always observed for , the occurrence of SSR in the STS model depends on the specific choice of the model parameters.

Using Eq. (44) and Eq. (48), we can find for  and  a noise strength 

for which the lower bound on the mutual information rate is equal for both models. From the above equation we can see that whether the STS population or the AD population transmits more information for a given value of independent noise is mainly determined by the noise and signal cutoff frequencies  and .

**Fig. 9:**
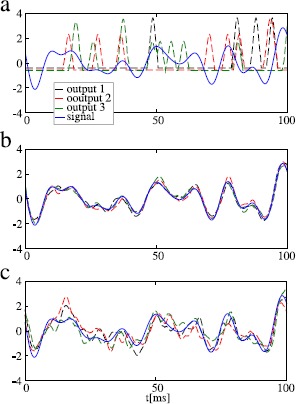
We illustrate the emergence of SSR in the STS model for the same parameters as in Fig. 6d. **a** A fixed realization of the signal (*blue*) and three realizations (different common noise realizations) of the output of the STS population for  and  (*black*, *green*, and *red*). For better visualization the output is convoluted with a Gaussian filter and all outputs and the signal are rescaled to unit variance and zero mean over the time window shown. For vanishing independent noise, the individual spike trains of the population are identical for a fixed realization of the signal and the common noise. In this case signal transmission is not improved by the large population size. **b** Same as in **a** but for  (close to the point of stochastic resonance). The individual noise leads to shifting of spikes, such that the convoluted summed output is smoothed. Note that the three realizations of the output are all close to the input signal as well as to each other, indicating a reliable signal transmission. **c** Same as in **a** but for  (far beyond the point of stochastic resonance). Note that our average over a comparatively short time window implies the suppression of long-term variability (corresponding to leaving out low-frequency components of the coherence function)

Finally, let us illustrate in Fig. 9 the stochastic resonance effect when it is most pronounced, namely, in the STS model for a large number of neurons () and a high cutoff frequency (except for *N*, all parameters as in Fig. 6d). In this situation, we consider the low-pass filtered summed output of the population for different levels of the intrinsic noise. Without intrinsic noise (Fig. 9a), the output, i.e. the sum of *N* perfectly synchronized spike trains, does not resemble the input signal very much. It is important to note that according to Eq. (19) and Eq. (9) an average over many such runs would yield a time series that tracks the input signal closely. However, single runs (red, black, green) in the absence of the intrinsic noise are strongly unreliable. The right amount of intrinsic noise (used in Fig. 9b) desynchronizes the *N* spike trains, reduces cross-correlations at high frequencies, and thus reduces output variability due to the common noise. Consequently, different realizations of the process for a frozen input signal look more similar and track the input signal reliably (cf. Fig. 9b). However, if we increase intrinsic noise to much higher levels, as in Fig. 9c, this noise itself starts to contribute significantly to the output variability and the reliability of signal transmission is diminished again.

## 6 Summary and Conclusions

In this paper, we investigated how the effect of noise on the output spikes influences information transmission properties of Poisson neurons. In particular, we considered two populations with strong common input, where in one case weak independent noise added and deleted spikes, while in the other it shifted spikes. In the limit of a weak sensory signal, we analytically derived the spectral statistics of both models and studied information filtering and the emergence of suprathreshold stochastic resonance (SSR). We showed that, even when single neurons of the AD model and STS model cannot be distinguished by their response statistics, the different effects of independent noise on spikes lead to qualitative and quantitative differences in information transmission on a population level.

In the AD model, the presence of the SSR effect is robust—whenever we consider a population with , a small amount of intrinsic noise has a beneficial effect on the signal transmission. In the STS model, the information transmission properties of the population are determined by the cutoff frequencies of the noise. Depending on the specific parameters, one finds a pronounced SSR in some regimes (exceeding the effect in the AD model by far) or no SSR effect in other regimes. Furthermore, we observe a high-pass filtering of information in the STS model that is absent in the AD model.

There are a number of studies that explored theoretically the case of weakly correlated neurons and employed perturbation methods to relate output spike train correlations to input correlations [46–52]. In this paper, we have considered the opposite limit of strongly correlated spike trains that are only weakly *decorrelated* due to intrinsic noise sources. In this limit, we were not only able to derive comparatively simple expressions for the cross-correlation between two spike trains but were also able to explore analytically the consequences of these correlations for the transmission of time-dependent signals.

The question arises how the specific choice of the output, which is taken to be the sum of individual spike trains, affects the findings discussed above. The most general approach would be to study the multivariate mutual information between the input signal and the population of output spike trains. This quantity is hard to compute numerically and analytically, and its exact calculation is beyond the scope of this study. However, the mutual information between the input signal and the sum of outputs is a lower bound for the full multivariate mutual information, because the summation can only degrade the information content contained in the entire set of the output spike trains. Additionally, for vanishing individual noise, , all output spike trains are identical and the information content of the population does not differ from the information content of the sum of identical spike trains. Therefore, if the mutual information between the input signal and the summed output increases with individual noise, i.e. exhibits suprathreshold stochastic resonance, the full multivariate mutual information increases as well.

The mutual information between the input signal and the summed output has been estimated here by its lower bound . In our setting with a weak signal that is encoded in the firing rate of the Poisson process, we expect that this bound is rather tight. In fact, for a single inhomogeneous Poisson process, the mutual information and its lower bound coincide in leading-order of the signal amplitude [53].

In this study, we inspected two simple and abstract models for the effect of a weak noise on neural spikes and its consequences on signal transmission by neural populations. We would like to emphasize that the pure limits of an AD model or an STS model approximate the behavior of biophysical neuron models. On one hand, it is plausible that in an excitable neuron model, in which the crossing of a threshold may be aided or prevented by a weak driving, addition and deletion of spikes as in our AD model can be observed. Stochastic oscillators, on the other hand, display a shifting of spike times due to a weak driving, as described by the phase response curve [54]. In between these limits, we expect a combination of both, addition and deletion as well as shifting of spikes. Indeed, such a combination has been observed experimentally [55]. Hence, a generalization of our framework to a Poisson process that includes both effects and allows one to tune gradually between the pure AD and STS models inspected in this paper would be certainly worth additional efforts in a future study.

## Appendix A: Mean Firing Rate of the AD and STS Model

For the AD model the average of the spike train over the intrinsic noise is given by Eq. (13) as 

The average of the spike train over all stochastic processes can now be written as 49

With  from Eq. (11) the first term in the above equation gives 50

as *s* and *η* are Gaussian processes with unit variance and zero mean. For the other terms in Eq. (49), we will show that they are of higher-order in Δ*t*, , and . For the second term in Eq. (49), we can find an upper bound using the Cauchy–Schwarz inequality 51

The average in the last line of the above equation is the probability that  is smaller than zero and is given by 52

which gives for Eq. (51) 53

For the third term in Eq. (49) we can find an upper bound using again the Cauchy–Schwarz inequality 54

where in the last line we have dropped the mixed term that is always negative. Furthermore, note that the average in the last line of Eq. (54) is the probability that  is larger than  and is given by 55

which gives for Eq. (54) 56

Inserting Eqs. (50), (53), and (56) into Eq. (49), we obtain the mean firing rate for the AD model 

In the limit of , the last term in the above equation can be dropped and we obtain 57

A similar estimation leads to the same formula for the STS model.

## Appendix B: Probability for Synchronous Spikes in the AD Model

For the AD model, according to Eq. (23), the probability to observe two spikes in a time window Δ*t* in two spike trains  and  is given by 

where  is a Gaussian distribution with unit variance and zero mean. Splitting the integration interval of the last integral in the above equation into two parts, such that for one interval  and for the other , we obtain 

which after a change of the order of integration can be transformed into 58

The average of the theta function over *ξ* reads 

Using the above equation, Eq. (58) can now be written as 

The order of the second and the third term in the above equation can be calculated analogously to the calculation in Appendix A which leads to 59

## Appendix C: Simplification of the Cross-correlation Function for the STS Model

In this section, we simplify Eq. (33) in Sect. 4.3. Therefore, we first consider 60

where the average over  is conditioned on fixed realizations of the signal  and the noise process . The variable  is defined in Eq. (32). We first express the delta function in the above expression as a derivative of a Heaviside function, which leads to 61

As we will show in Eq. (63), the variance  becomes constant in the limit of large . Performing the derivative with respect to  in Eq. (61), we find 

Combining the above equation with Eq. (60), we finally obtain the relation 

Using the above result, we rewrite the average over the delta function in Sect. 4.3 in Eq. (33) as 

Expressing the delta function in the above equation as the derivative of a Heaviside function with respect to  as in Eq. (61) and following the steps from the calculation of , we find 

which leads to the result 62

used in the calculation of the cross-correlation function in Eq. (34).

## Appendix D: Variance of the Integrated Independent Noise

To calculate the variance from Eq. (35), we first consider 

for a Gaussian signal  with zero mean and unit variance. Performing the average in the above equation and performing a change of integration variables, we can write 

Next, we express the autocorrelation function in the above equation by its Fourier transform according to Eq. (6) and find 

where in the last line of the above equation we integrated over *τ*. Since the power spectrum  is the Fourier transform of a real function, it is symmetric with respect to *f*, which leads to 

For a band-pass limited white noise with the power spectrum 

we obtain in the limit of large times 63

and for the variance in Eq. (35) for large *t* we find 64

## Appendix E: Single Spike Train Power Spectrum

In this section, we calculate the single spike train power spectrum for the AD model. Therefore, we consider the autocorrelation function of the spike train 65

where we assume that the spike trains are stationary. Since a single spike train is considered and the average is taken over one independent noise process *η*, we will drop the subscript employed previously.

We first consider the AD model. As for the time-discrete cross-correlation function between two spike trains in Sect. 4.2, we can express Eq. (65) in terms of probability densities 66

where  is the probability to find a spike in a given time bin of width Δ*t* and  is the probability to find two spikes separated by the time interval *τ* with . Analogously to Eq. (23) we find 

where in the first line of the above equation we dropped the subscript and the time-argument for the parameter *r*, which is defined in Eq. (11). For the probability of asynchronous spikes with  we find 

which in the limit of  leads for the autocorrelation function Eq. (66) to 

Taking the limit  in the above equation (keeping terms up to second-order in  and ), we find after a Fourier transformation the single spike train power spectrum for the AD model 67

Next, we calculate the single spike train power spectrum for the STS model. Employing Eq. (17), the last term in Eq. (65) can be written as 68

In analogy to Eq. (33), we can transform the first term of the autocorrelation function Eq. (65) into 

with the difference that we now consider a single spike train, and therefore only averages over one independent noise process *η*. The above equation can be rewritten as 69

After a Fourier transformation of the above equation, the autocorrelation functions of signal and noise are transformed into their respective power-spectra. Inserting the definition of  Eq. (32), the first term of Eq. (69), which we denote by *W*, is Fourier transformed into 70

Changing the integration variable in Eq. (70), we find 

For a strictly positive process  (see Eq. (11)), the only zero crossing of the integral  is at . We can invert this relation to find , which leads to 71

The order of the correction term in the above equation is proportional to the square root of the probability that , which has been calculated in Appendix A Eq. (52).

Employing Eqs. (65), (68), (69), and (71) we find in the limit  (keeping terms up to second-order in  and ) the single spike train power spectrum for the STS model 72
